# Overcoming barriers to vaginal hysterectomy: An analysis of perioperative outcomes

**DOI:** 10.4274/jtgga.galenos.2018.2018.0021

**Published:** 2019-02-26

**Authors:** Ido Sirota, Shannon A. Tomita, Lisa Dabney, Alan Weinberg, Linus Chuang

**Affiliations:** 1 Department of Obstetrics and Gynecology, New York-Presbyterian Queens Weill Cornell Medicine, New York, USA; 2 Department of Obstetrics, Gynecology and Reproductive Sciences, Icahn School of Medicine at Mount Sinai, New York, USA; 3 Department of Genetics and Genomic Sciences, Icahn School of Medicine at Mount Sinai, New York, USA; 4 Department of Population Health Science and Policy, Icahn School of Medicine at Mount Sinai, New York, USA; 5 Department of Obstetrics and Gynecology, Danbury Hospital – Western Connecticut Health Network, Connecticut, USA

**Keywords:** Vaginal hysterectomy, perioperative outcomes, minimally invasive

## Abstract

**Objective:**

To determine perioperative outcome differences in patients undergoing vaginal hysterectomy based on uterine weight, vaginal delivery, and menopausal state.

**Material and Methods:**

Retrospective chart review of 452 patients who underwent vaginal hysterectomy performed by a single surgeon. Patients’ age, vaginal delivery, uterine weight, previous pelvic surgery, previous cesarean delivery, removal of ovaries were compared, as well as estimated blood loss (EBL), operating room time (ORT), length of stay, intraoperative complications and postoperative complications. Multivariable logistic regression was used, and all data were analyzed at the level of p<0.05 statistical significance using SAS system software (SAS Institute Inc., Cary, NC), version 9.3.

**Results:**

The mean age was 57.13±11.52 years and the median vaginal delivery was 2. The uterine weight range was 16.6-1174.5 g (mean 169.79±183.94 g). The incidences of blood transfusion and bladder injury were 3.03% and 0.66%, respectively. Factors shown to be associated with longer ORT included greater uterine weight, removal of ovaries, posterior repair, tension-free vaginal tape sling, prolapse, and EBL >500 mL (p<0.001). The factors associated with EBL >500 mL were greater uterine weight (p=0.001), uterine myomas (p=0.016) and premenopausal state (p=0.014). The factors associated with conversion to laparotomy were greater uterine weight (p<0.001) and premenopausal state (p<0.001).

**Conclusion:**

Vaginal hysterectomy is a safe and feasible approach for patients desiring hysterectomy regardless of uterine weight and vaginal delivery.

## Introduction

Hysterectomy is the most frequent non-pregnancy-related major surgical procedure performed on women in the United States (US) (1,2). The most common benign indications include leiomyomas, endometriosis, and prolapse, followed by pelvic pain, dysfunctional uterine bleeding, adenomyosis, pelvic inflammatory disease, and obstetric indications (1,2). Evidence suggests that when deemed feasible, the vaginal approach is the safest route of performing hysterectomy for benign disease and is considered the gold standard approach (2,3,4,5,6,7,8). When compared with abdominal hysterectomy, it is associated with fewer complications, including urinary tract injury and infection, as well as better economic outcomes and perioperative outcomes including operating room time (ORT), length of hospital stay, and recovery time (3,4,5,6,7,8). In addition, multiple studies have shown no benefit of laparoscopic-assisted vaginal hysterectomy when compared with vaginal hysterectomy (9). Furthermore, vaginal hysterectomy was associated with shorter operative time and shorter hospital stay compared with total laparoscopic hysterectomy and laparoscopic-assisted vaginal hysterectomy (9). 

Several studies comparing perioperative outcomes in vaginal hysterectomy versus robotically-assisted laparoscopic hysterectomy found that vaginal hysterectomy was associated with shorter operative time, overall comparable perioperative outcomes, and lower cost (10,11,12,13,14,15). Jacome et al. (11) reported a slight increase in “major” intraoperative complications with vaginal compared with the robotic surgical approach, although those “major intraoperative complications” were never defined and the statistical analysis was underpowered. Additionally, a study by Martino et al. (16) reported lower estimated blood loss (EBL), shorter hospital length of stay, and lower readmission rates in robotic-assisted laparoscopic hysterectomies when compared with all non-robotic surgical approaches including vaginal hysterectomy in their retrospective study of 2554 patients. Even though the American College of Obstetricians and Gynecologists has considered vaginal hysterectomy as the gold standard approach, vaginal hysterectomy is still largely underutilized as evidenced by US statistics, which show that 66% of hysterectomies performed for benign conditions are still being performed by the abdominal route, 22% vaginally and, 12% laparoscopically, with the rate of vaginal hysterectomy having steadily declined since its peak in 2002 (2,17). 

Historically, physicians have used certain clinical criteria to exclude patients as candidates for vaginal hysterectomy, including large uterine size, a narrow vagina or narrow pubic arch, prior pelvic or abdominal surgery, and undescended or non-mobile uterus (18,19,20). Currently, there is a growing body of evidence that such rigid guidelines should not be used to limit the use of vaginal hysterectomy. Multiple studies have shown high success rates performing vaginal hysterectomy despite enlarged uteri (3,17,21,22). Furthermore, nulliparity has also been largely dispelled as a potential barrier for success of vaginal hysterectomy (2,5,6,8,17,23). Nulliparity used in this context refers to anatomic considerations such as narrow vaginal introitus, narrow pubic arch and/or lack of descensus, which have traditionally been associated with nulliparity. 

Our study aims to examine factors associated with successful vaginal hysterectomy despite perceived challenges based on patient history and physical examination. We sought to determine if any differences in perioperative outcomes existed in patients undergoing vaginal hysterectomy based on uterine weight, parity, and menopausal state. We hypothesized that given an experienced surgeon, vaginal hysterectomy should remain the gold standard approach regardless of large uterus, nulliparity or menopausal status.

## Material and Methods

This is a retrospective descriptive study of a prospectively collected database, with 452 patients’ charts reviewed in total. All patients underwent vaginal hysterectomy by a single urogynecologist with a referral-based practice in a community academically-affiliated hospital between March 2003 and March 2016. The surgeon was assisted on all cases by OB/GYN residents in training. The most common indications for surgery included uterovaginal prolapse, abnormal uterine bleeding, and uterine leiomyoma, which accounted for 94% of the surgical indications.

Vaginal hysterectomy was performed by first entering the posterior cul de sac sharply followed by progressive clamping of the suspensory ligaments (uterosacral, cardinal, uteroovarian) with curved Heaney clamps and ligating with Vicryl sutures on both sides. McCall’s culdoplasty was performed on all patients prior to closure of the vaginal cuff by placating the uterosacaral ligaments to the peritoneal surface prior to vaginal cuff closure. If salpingoophorectomy was performed, it was done so by grasping the adnexa with a Babcock clamp and using a Vicryl Endoloop to ligate the pedicle followed by transection of the adnexa.

Information was extracted from pre-operative patient histories, pathology reports, and operative reports. Variables studied include patient age, vaginal delivery, uterine weight, indication for surgery, previous pelvic surgery, previous cesarean delivery, and removal of ovaries. These variables were examined for statistically significant associations with perioperative complications, our primary outcome. For our study, we defined patients with uterine weight >250 g as having a “large uterus”. Menopausal status was determined by patient history, referring to the absence of menses >1 year. Perioperative complications in our study were defined as EBL more than 500 mL, conversion to laparotomy, ureteral and bladder injuries, as well as post-operative complications during the 6 weeks following surgery, which includes bowel injury, vaginal cuff cellulitis, pelvic collections/abscesses, ureteral injury, bladder injury, and post-op fever.

All factors were explored using a multivariable logistic regression. For ORT analysis, multiple linear regression was used. All data were analyzed at a level of p<0.05 statistical significance using SAS system software (SAS Institute Inc., Cary, NC), version 9.3.

## Results

The study population included a total of 452 patients. The mean age was 57.13±11.52 (range, 26-85) years, and the median number of vaginal deliveries was 2 (range, 0-8) (Table 1). The uterine weight range was 16.6-1174.5 g with a mean of 169.79±183.94 g (Table 1). The overall incidence of blood transfusions and bladder injuries were 3.03% and 0.66%, respectively (Table 3, 4, 5). Seven patients were converted to abdominal hysterectomy with a conversion rate of 1.5% (Table 2). The factors associated with conversion to laparotomy were greater uterine weight (p<0.001) and premenopausal state (p<0.001). Conversion to laparoscopy was not chosen in these 7 cases due to physician preference. As stated above, all patients undergoing vaginal hysterectomy had uterosacral suspensions, including those who did not have hysterectomies for prolapse. There were 2 readmissions within 6 weeks post-op, including one for a bowel injury and one for a pelvic abscess. The patient with the bowel injury had history of multiple prior laparotomies and although the hysterectomy was able to be completed vaginally, the patient re-presented 2 week later with peritonitis and was found to have a small enterotomy in the sigmoid colon.

The factors associated with longer ORT were uterine weight, removal of ovaries, posterior repair, tension-free vaginal tape sling, prolapse, and EBL more than 500 mL (p<0.001). The factors associated with EBL more than 500 mL were uterine weight (p=0.001), uterine myomas (p=0.016), and premenopausal state (p=0.014). No significant difference was noted in the incidence of blood transfusions, bladder and ureteral injuries, as well as readmissions in patients regardless of uterine weight, vaginal delivery or menopausal status (Table 3, 4, 5).

## Discussion

The objective of our study was to determine if perioperative differences existed in patients undergoing vaginal hysterectomy based on uterine weight, vaginal delivery, and menopausal state. We found that although greater uterine weight was associated with longer ORT and EBL more than 500 mL, no significant differences were noted in the incidence of blood transfusions, bladder/ureteral injury, or readmissions in patients regardless of uterine weight, vaginal delivery or menopausal state (Table 3, 4, 5). Although conversion to laparotomy was found to be associated with greater uterine weight, the overall incidence of conversion was 1.5%, which is exceedingly low for a cohort of this size. Our findings are in agreement with the current literature, which emphasizes that vaginal hysterectomy can be successfully performed with favorable perioperative outcomes in patients with characteristics previously perceived to be contraindications. 

Benassi et al. (3) examined uterine weight as a risk factor for perioperative complications in patients undergoing vaginal hysterectomy (n=60) versus abdominal hysterectomy (n=59) for fibroid uteruses weighing 200-1300 g. The results were shorter ORT, shorter hospital stay, as well as lower incidence of post-operative fever and demand for post-operative analgesics in the vaginal hysterectomy group compared with the abdominal hysterectomy group. Darai et al. (22) similarly evaluated uterine weight as a risk factor for successful completion of vaginal hysterectomy. Eighty patients who were referred for abdominal hysterectomy were randomized to vaginal hysterectomy vs laparosopic-assisted vaginal hysterectomy. The inclusion criteria included uterine weight more than 280 g, as well as one or more commonly considered contraindications to vaginal hysterectomy such as prior pelvic surgery, history of pelvic inflammatory disease, moderat-to-severe endometriosis, adnexal masses or nulliparity without uterine descent. The investigators found that the complication rates were lower in the vaginal hysterectomy group compared with the laparoscopic-assisted vaginal hysterectomy group (15% and 37%, respectively) and vaginal hysterectomy was associated with shorter ORT.

Agostini et al. (23) examined nulliparity as a risk factor for increased perioperative complications. The study included 345 women without uterovaginal prolapse and without prior pelvic surgery undergoing vaginal hysterectomy for benign indications. Only 52 patients were nulliparous; however, the authors concluded that ORT and overall complication rate was higher in nulliparous patients. Additionally, Tohic et al. (24) evaluated 300 patients without previous vaginal delivery for success rates of planned vaginal hysterectomy and found a 92.1% success rate; however, perioperative outcomes were not reported. In another study by Harmanli et al. (19) 75 women with the intention of undergoing vaginal hysterectomy by a single surgeon were included in the study. Fifty patients successfully underwent vaginal hysterectomies compared with 25 that failed. Although multiple factors were compared between the two groups, the investigators concluded that the only patient characteristic associated with an increased risk of failure for vaginal hysterectomy was the presence of a narrow pubic arch, which was determined clinically by the surgeon. 

Figueiredo et al. (5) evaluated perioperative outcomes in 300 women without prolapse undergoing vaginal hysterectomy for benign indications. Vaginal delivery and prior pelvic surgery were the only risk factors evaluated, and only 7% of their cohort was nulliparous. They similarly concluded no significant differences in perioperative outcomes.

Doucette et al. (4) examined vaginal hysterectomy success rates and perioperative complications in a group of 250 patients with large uterus weighing more than 180 g, and either no prior vaginal delivery or previous cesarean section or pelvic laparotomy. The study had three control groups that underwent either laparoscopic-assisted vaginal hysterectomy (n=250), vaginal hysterectomy (n=250) or abdominal hysterectomy (n=250). They concluded that large uterus, nulliparity, previous cesarean delivery, and pelvic laparotomy rarely constituted contraindications to vaginal hysterectomy, and vaginal hysterectomy was found to be associated with the least number of perioperative complications when compared with the laparoscopic-assisted vaginal hysterectomy and abdominal approaches.

In a study by Paparella et al. (20) the investigators prospectively enrolled 204 patients with benign indications for hysterectomy to undergo vaginal hysterectomy by a single experienced vaginal surgeon with an experienced laparoscopic surgeon available if needed for laparoscopic assistance or conversion. Each patient had one or more commonly considered contraindications to vaginal surgery, including prior pelvic surgery, history of pelvic inflammatory disease, moderate-to-severe endometriosis, adnexal masses or nulliparity with lack of uterine descent, and limited vaginal access. Patients were thus divided into five groups, corresponding to each of the commonly considered contraindications listed above. The perioperative factors being evaluated were identical to those evaluated in our study. Similarly to our study, they found no statistically significant differences in complication rates among the five groups of patients studied. However, this study excluded patients with prolapse. Two major limiting factors of this study were the lack of a control group for comparison of perioperative outcomes and the small patient sample size, which was less than half of that presented in our study.

Lastly, in addition to the literature comparing perioperative outcomes in patients with different pre-operative characteristics undergoing vaginal hysterectomy, there is also a substantial amount of literature comparing vaginal hysterectomy with other minimally invasive hysterectomy approaches such as laparoscopic hysterectomy, laparoscopic-assisted vaginal hysterectomy, and robotic-assisted laparoscopic hysterectomy (4,10,11,12,13,14,15,16,21,22,25,26). The conclusions of these studies are mixed with regard to comparison of perioperative outcomes; however, it has consistently been noted that robotic-assisted laparoscopic hysterectomy is associated with longer ORT and higher costs of care.

The strengths of our study include the large cohort of cases over an extended period of time performed by the same surgeon with a wide variety of patient characteristics and outcomes examined. Currently, our study is the largest that we know of that evaluates and dispels multiple patient characteristics as risk factors for vaginal hysterectomy as opposed to other studies that mainly examined a single risk factor (3,19,22,23). Our findings are limited by the retrospective nature of the study, the lack of power given that many outcomes were infrequent, and lack of Pelvic Organ Prolapse Quantification scores, which prevented us from identifying the degree of prolapse in each patient studied. In addition, the fact that all cases were performed by a single surgeon limits the ability to generalize the results to all surgeons, because the results of a highly experienced surgeon are not likely to be replicated. Lastly, the surgeon being assisted by different residents in each case is also a limitation, given that assistance by a senior compared with a junior resident may have theoretically resulted in better outcomes.

In conclusion, we believe our study supports the literature that vaginal hysterectomy is a feasible and safe approach despite commonly perceived challenges to its success. We have demonstrated favorable and comparable perioperative outcomes in patients undergoing vaginal hysterectomy regardless of uterine size, vaginal delivery or menopausal status. Although barriers to increased use of vaginal hysterectomy have been identified, further randomized controlled trials are needed to evaluate the feasibility and efficacy of various interventions proposed to increase the use of the vaginal approach.

## Figures and Tables

**Table 1:**
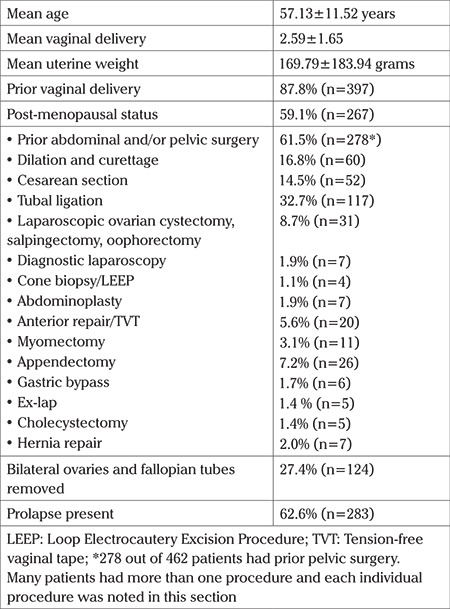
Patient demographics and characteristics

**Table 2:**
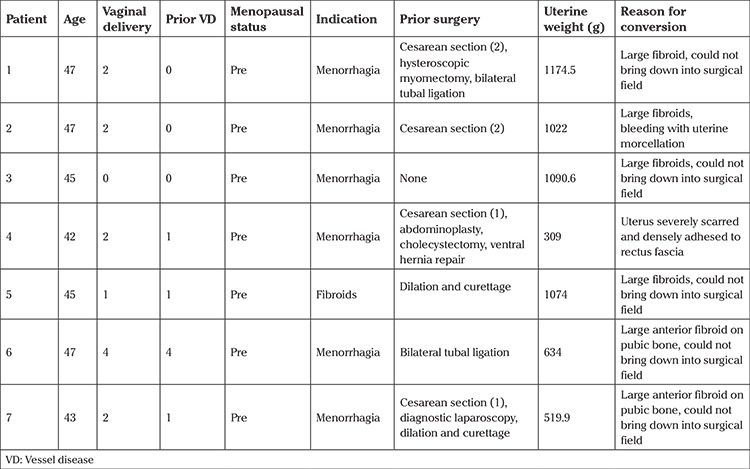
Clinical information for patients converted to laparotomy

**Table 3:**
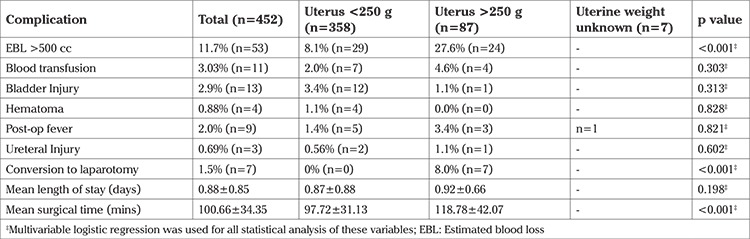
Perioperative complications and uterine weight

**Table 4:**
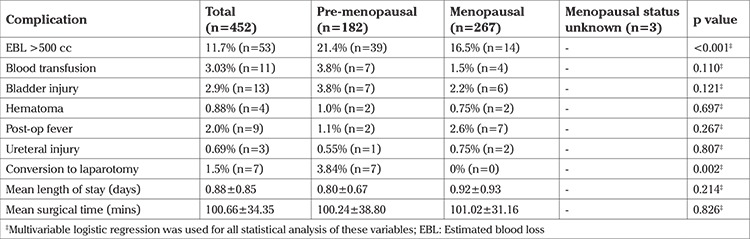
Perioperative outcomes and menopausal status

**Table 5:**
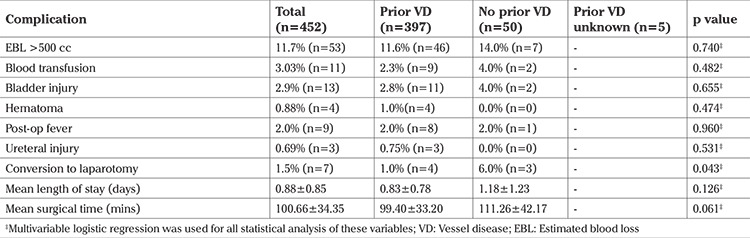
Perioperative complications and prior vaginal delivery
